# Antimicrobial resistance among producers and non-producers of extended spectrum beta-lactamases in urinary isolates at a tertiary Hospital in Tanzania

**DOI:** 10.1186/1756-0500-3-348

**Published:** 2010-12-24

**Authors:** Sabrina J Moyo, Said Aboud, Mabula Kasubi, Eligius F Lyamuya, Samuel Y Maselle

**Affiliations:** 1 Department of Microbiology and Immunology, Muhimbili University of Health and Allied Sciences, Dar es Salaam, Tanzania; 2 Department of Laboratory Services-Central Pathology Laboratory, Muhimbili National Hospital, Dar es Salaam, Tanzania

## Abstract

**Background:**

Published data on the existence and magnitude of extended spectrum beta-lactamase (ESBL) production in urinary pathogens in local setting is limited. The aim of the present study was to determine the prevalence of antimicrobial resistance and ESBL production among *Escherichia coli *and *Klebsiella spp *from urine samples in a tertiary hospital. This was a cross sectional study conducted at Muhimbili National Hospital in Dar es Salaam, Tanzania.

**Findings:**

A total of 270 *E.coli *and *Klebsiella spp *urinary pathogens from children and adults isolated from January to March 2010 were included in the study. *E. coli *and *Klebsiella spp *isolates were tested for antimicrobial susceptibility by the Clinical and Laboratory Standard Institute's disc diffusion method. These isolates were further screened for ESBL phenotype using cefotaxime and ceftazidime discs. Isolates with reduced sensitivity were confirmed using ESBL E-test strips. Of 270 isolates, 138 (51.1%) were *E. coli *and 132 (48.9%) were *Klebsiella spp*. ESBL was detected in 122 (45.2%) of all the isolates. ESBL- producing *E. coli *strains were significantly more resistance to cotrimoxazole (90.7%), ciprofloxacin (46.3%) and nalidixic acid (61.6%) than strains that did not produce ESBL (p < 0.05). Similarly, ESBL- producing *Klebsiella spp *strains were significantly more resistance to cotrimoxazole (92.6%), ciprofloxacin (25.0%), nalidixic acid (66.2%), and gentamicin (38.2%) than strains that did not produce ESBL (P < 0.05). Multi-drug resistance was found to be significantly (*P *< 0.05) more in ESBL producing isolates (90.5%) than non ESBL producers (68.9%). The occurrence of ESBL was significantly higher among isolates from inpatients than outpatients [95 (50.5%) vs. 27(32.9%)] (p = 0.008). The occurrence of ESBL was significantly higher among isolates from children than in adults [84 (54.9%) vs. 38(32.5%)] (p < 0.001).

**Conclusions:**

High prevalence of ESBL-producing *E. coli *and *Klebsiella spp *strains was found among inpatients and children. Most of the ESBL- producing isolates were multi-drug resistant making available therapeutic choices limited. We recommend continued antibiotic surveillance as well comprehensive multi-center studies to address the emerging problem of ESBL-associated infections in order to preserve the continued usefulness of most antimicrobial drugs. Further more conducting molecular studies will help to evaluate the various ESBL types.

## Background

Urinary tract infection (UTI) is the second most common infectious presentation in community practice. Worldwide, about 150 million people are diagnosed with UTI each year, costing the global economy in excess of 6 billion US dollars [1]. There have been significant changes in the antimicrobial resistance patterns of uropathogens over the years including resistance due to extended spectrum beta lactamase (ESBL)-producing pathogens [2-6]. The increasing prevalence of infections caused by antibiotic-resistant bacteria makes empirical treatment of these infections difficult [7]. Antibiotic resistance varies according to geographic locations and is directly proportional to the use and misuse of antibiotics. Understanding the impacts of drug resistance is crucial as the changing rate of antibiotic resistance has a large impact on the treatment of UTI [3,4].

Production of ESBL is the most common amongst the mechanisms of resistance to third generation cephalosporins in Gram-negative bacilli [8]. While there are many published reports on ESBL-producing microorganisms in developed and developing countries [5,6,9], two previous studies which were done in 2005 confirmed the presence of ESBL-producing organisms in nosocomial and blood stream infections in a tertiary referral hospital in Dar es Salaam [10,11]. The current study aimed to determine the prevalence of antimicrobial resistance and ESBL production among *E. coli *and *Klebsiella spp *isolated from patients with UTI in a tertiary hospital.

## Materials and methods

### Study design and setting

This was a cross-sectional study conducted at Muhimbili National Hospital (MNH) in Dar es Salaam, Tanzania. Urinary isolates collected from January to March 2010 were included in the study.

### Bacterial isolates

Urine samples received in the Central Pathology Laboratory (CPL) were plated on cysteine lactose electrolytes deficient (CLED) agar incubated at 37°C for 24 hours. A growth of >10^5 ^colony forming units per mL of one type of organism was considered as significant bacteriuria. Identification of *E. coli *and *Klebsiella spp *isolates was done by observing colonial morphology on CLED medium. Lactose-fermenting colonies were further identified using standard biochemical tests as described previously [12].

### Antibiotic susceptibility testing

*E. coli *and *Klebsiella spp *isolates were tested for antimicrobial susceptibility by the Clinical and Laboratory Standards Institute's disc diffusion method, was formerly the National Committee for Clinical Laboratory Standards [13]. The following first and second line antibiotics were used in the testing: ceftazidime (30 μg), cefotaxime (30 μg), cotrimoxazole (25 μg), ampicillin (10 μg), gentamicin (15 μg), amikacin (30 μg), imipenem (10 μg), ciprofloxacin (5 μg), nalidixic acid (30 μg), and nitrofurantoin (300 μg). Multi-drug resistance was defined as resistance to three or more different antimicrobial agents.

### Detection of ESBL

*E. coli *and *Klebsiella spp *isolates were screened for ESBL phenotype. Isolates with reduced susceptibilities to cefotaxime (zone diameter of < 27 mm) and/or ceftazidime (zone diameter of < 22 mm) were provisionally regarded as ESBL-producing pathogens according to guidelines for laboratory detection of ESBL from Clinical and Laboratory Standards Institute [13]. ESBL E-test strips (Biomerieux, Solna, Sweden) were used for confirmation of ESBL production. Minimum inhibitory concentrations (MIC) of cefotaxime and ceftazidime with and without clavulanic acid were determined. The inoculated plates were incubated for 16-18 hours at 37°C.

ESBL results were read either as MIC values or observation of 'phantom' zones or deformation of inhibition ellipses according to manufacturer instructions. Reduction of MIC by at least three two-fold dilutions in the presence of clavulanic acid was indicative of ESBL production. Deformation of ellipses or the presence of a 'phantom' zone was also indicative of ESBL production even if the MIC ratio was <8 or could not be read. Isolates were reported as having ESBL phenotype if one or more of the ESBL E-tests were positive. *E. coli *ATCC 25922 was used as ESBL negative control and *K. pneumoniae *ATCC 700603 was used as ESBL positive reference strain.

### Statistical analysis

Statistical Package for the Social Sciences (SPSS) version 17.0 was used for data analysis. Contingency table analysis was done by a chi-square test or two-tailed Fisher's exact test where applicable. A p-value of less than 0.05 was considered as statistically significant.

### Ethical considerations

The study was carried out in accordance with existing ethical guidelines. Ethical clearance was obtained from the Senate Research and Publications Committee of Muhimbili University of Health and Allied Sciences, Dar es Salaam.

## Results

During the study period, a total of 270 *E. coli *and *Klebsiella spp *were isolated. The urinary pathogens were isolated more from female (54.4%) than male (45.6%) patients (Table 1). Of the total number of bacterial isolates obtained 56.7% were from paediatric patients while 43.3% were from adults. Of the 270 isolates, 138 (51.1%) were *E. coli *and 132 (48.9%) were *Klebsiella spp*. There was significantly higher proportion of bacteria isolated from inpatients (69.6%) than outpatients (30.4%) (p = 0.008).

**Table 1:** Distribution of bacteria isolates according to patient age and sex, wards and bacterial species (n = 270)

Characteristics	Number	Percentage
**Age**		
Children	153	56.7
Adults	117	43.3
**Sex**		
Male	123	45.6
Female	147	54.4
**Wards**		
Out patients	82	30.4
In patients	188	69.6
**Pathogens isolated**		
*E. coli*	138	51.1
*Klebsiella *spp	132	48.9
ESBL producing *E. coli *(n = 138)	54	39.1
ESBL producing *Klebsiella spp *(n = 132)	68	51.5

The frequency of antimicrobial resistance for 11 selected antimicrobial agents against *E. coli *and *Klebsiella spp *UTI pathogens are summarized in Table 2 and 3. ESBL was detected in 122 (45.2%) of all the isolates. Among *E. coli *isolates 39.1% were ESBL- producers. *E. coli *showed high rate of resistance was seen to ampicillin (96.4%), followed by cotrimoxazole (80.4%) and amoxicillin/clavulanic acid (69.6%) (Table 2). Resistance to cefotaxime and ceftazidime was 50.7% and 49.3%, respectively. *E. coli *showed showed least rate of resistance to imipenem (6.5%). ESBL- producing *E. coli *were significantly more resistant to cotri-moxazole (90.7%), ciprofloxacin (46.3%) and nalidixic acid (61.6%) than non-ESBL *E. coli *producing strains (p < 0.05). Multi-drug resistance was significantly higher among ESBL- producing *E. coli *strains than non-ESBL producing *E. coli *(87.0% vs. 54.8%) p < 0.001.

**Table 2:** Antimicrobial resistance of ESBL and non-ESBL producing E. coli in Dar es Salaam, Tanzania (n = 138)

Antibiotic	Resistance n (%)	ESBL- Producing n = 54	Non-ESBL producing n = 84	P-value*
Cefotaxime	70(50.7%)	50(92.6)	20(23.8)	< 0.001
Ceftazidime	68(49.3%)	51(94.4)	17(20.2)	< 0.001
Ampicillin	133(96.4%)	53(98.1)	80(95.2)	0.37
Amoxycillin/ clavulanic acid	96(69.6%)	39(72.2)	57(67.9)	0.6
Imipenem	9 (6.5%)	4(7.4)	5(0.001)	0.7
Amikacin	15(10.9%)	6(11.1)	9(10.7)	0.9
Gentamicin	53(38.4%)	26(48.1)	27(32.1)	0.06
Nalidixic acid	57(41.3%)	28(61.6)	29(34.5)	0.04
Ciprofloxacin	42(30.4%)	25(46.3)	17(20.2)	0.001
Nitrofurantoin	31(22.5%)	11(20.4)	20(23.8)	0.6
Cotrimoxazole	111(80.4%)	49(90.7)	62(73.8)	0.014
Multi drug resistance	93 (67.4%)	47(87.0)	46(54.8%)	< 0.001

*P value is for a comparison of resistance among ESBL-producers with that among non-producers.

**Table 3:** Antimicrobial resistance of ESBL and non-ESBL producing Klebsiella spp in Dar es Salaam, Tanzania (n = 138)

Antibiotic	Resistance n (%)	ESBL- Producing n = 68	Non-ESBL producing n = 64	P-value*
Cefotaxime	87(65.9%)	68(100)	19(29.7)	< 0.001
Ceftazidime	87(65.9%)	68(100)	19(29.7)	< 0.001
Ampicillin	130(98.5%)	67(98.5)	63(98.4)	0.9
Amoxicillin/ clavulanic acid	103(78.0%)	57(83.8)	46(71.9)	0.09
Imipenem	9(6.8%)	6(8.8)	3(4.7)	0.2
Amikacin	28(21.2%)	17(25.0)	11(17.2)	0.2
Gentamicin	38(28.8%)	26(38.2)	12(18.8)	0.008
Nalidixic acid	65(49.2%)	45(66.2)	20(31.2)	< 0.001
Ciprofloxacin	25(18.9%)	17(25.0)	18(28.1)	0.04
Nitrofurantoin	24(18.2%)	14(20.6)	11(17.2)	0.5
Cotrimoxazole	109(82.6%)	63(92.6)	46(71.9)	0.001
Multi drug resistance	79(59.8%)	56(82.4)	23(35.9)	< 0.001

*P value is for a comparison of resistance among ESBL-producers with that among non-Producers.

Among isolates of *Klebsiella spp*, 51.1% were ESBL- producers. *Klebsiella spp *showed high rate of resistance to ampicillin (98.5%), followed by cotrimoxazole (82.6%) and amoxicillin/clavulanic acid (78.0%) (Table 3). Resistance to cefotaxime and ceftazidime was 65.9% and 65.9%, respectively. *Klebsiella spp *showed least rate of resistance to imipenem (6.8%). ESBL- producing *Klebsiella spp *strains were significantly more resistant to cotrimoxazole (92.6%), ciprofloxacin (25.0%), nalidixic acid and gentamicin (38.2%) than non-ESBL producing strains (p < 0.05). Other antimicrobial agents for which ESBL-producing strains showed significant resistance included cefotaxime (100%), ceftazidime (100%), cotrimoxazole (92.6%), ciprofloxacin (25.0%), nalidixic acid (66.2%) and gentamicin (38.2%) (p < 0.05). Multi-drug resistance was significantly higher among ESBL- producing *Klebsiella spp *strains than non-ESBL producing *Klebsiella spp *(82.4% vs. 35.9%) p < 0.001.

The occurrence of ESBL was found to be significantly higher among isolates from inpatients than outpatients [95 (50.5%) vs. 27(32.9%)] (p = 0.008). The rate of ESBL production was also significantly higher among isolates from children than in adults [84 (54.9%) vs. 38 (32.5%)] (p < 0.001).

## Discussion

Knowledge on local antimicrobial resistance trends among urinary isolates is important not only in guiding clinicians to prescribe appropriate antibiotics but also for evidence based recommendations in empirical antibiotic treatment of UTI. The current study described the antimicrobial resistance rates including detection of ESBL among *E. coli *and *Klebsiella spp *urinary isolates which are the predominant ESBL- producers.

In the current study the antimicrobial resistance rate of both *E. coli *and *Klebsiella spp *isolates was high to the first line antimicrobial agents such as ampicillin, cotrimoxazole, and amoxicillin/clavulanic acid. High resistance to first line drugs found in the current study is similar to other studies in developing countries [11,14,15]. The observation may be due to wide use of these drugs empirically because they are relatively cheap and also by being oral antibiotics they are easy to administer. In addition, resistance to cotrimoxazole may be due to the fact that this drug is widely used for prophylaxis against opportunistic infections associated with HIV whose prevalence in our setting is high. The high level of resistance to amoxicillin/clavulanic acid is in keeping with the previous report in the same hospital[11] as well as studies conducted in other countries [16]. These findings suggest that beta-lactam/beta-lactamase inhibitor combination may not be useful in our setting for the treatment of UTI. A previous study has documented treatment failures due to the use of beta-lactam/beta-lactamase inhibitor combinations for infections caused by ESBL-producing organisms [17].

We demonstrated a high prevalence of ESBL production by *E. coli *(39.1%) and by *Klebsiella spp *(51.5%) urinary isolates at MNH. These findings are higher than those reported five years ago in the same hospital[10,11]. In the previous studies, 25% and 17% of *E. coli *and *Klebsiella spp*, respectively, isolated from septicaemic patients [10] and 28.2% of enteric pathogens from intensive care unit (ICU) patients [11] were ESBL-producers. Several possible reasons may have contributed to the high prevalence of ESBL-producing urinary isolates. Since UTI is relatively common, wide spread use of broad-spectrum antibiotics due to inappropriate prescribing practices are most likely. In addition, the hospital does not perform routine screening of ESBL production in clinical isolates and hence there are no guidelines for isolation of patients carrying these ESBL- producing strains which could have resulted in the spread of these strains.

ESBL-producing *E. coli *and *Klebsiella spp *in this study showed a significantly high rate of resistance to non-beta lactam antibiotics. These findings are similar to those reported by others [11,18-20]. This observation may be explained by the fact that ESBL are plasmid-mediated enzymes which are transferable between one bacterium to another and such transferable plasmids also code for resistance determinants to antimicrobial agents other than beta-lactams [21]. ESBL-producing *E. coli and Klebsiella spp *showed significantly high resistance to ciprofloxacin. These findings are higher than the resistance found by Blomberg *et al *in the same study setting [10,11]. The increased resistance implies that ciprofloxacin should be used with caution.

Our findings show that carbapenem are the most effective and drug of choice against both ESBL and non ESBL- producing *E. coli *and *Klebsiella spp *as more than 90% of isolates were sensitive to imipenem. Although imipenem showed high rate of sensitivity; 6.5% and 6.8% isolates of *E. coli *and *Klebsiella spp*, respectively, were resistant. Reduced imipenem susceptibility has been described in *E. coli *with CTX-M-type ESBL [22]. CTX-M type ESBL has been described before in our settings [10,11], which in part could explain the observation in the current study. Therefore further studies are needed for molecular characterization of ESBL isolates circulating in our setting. With sensitivity rate of 79% for ESBL-producing isolates nitrofurantoin might be the only useful oral antibiotic in the treatment of UTI in the local setting, at least in a tertiary health facility.

There was significantly higher proportion of ESBL-producing isolates among isolates from in-patients than out-patients (p < 0.05). This finding is similar to that observed by Khanfar *et al *[16] and points to the possibility of nosocomial acquisition of UTI due to ESBL pathogens. These findings have significant implications for empirical management of patients with UTI using third generation cephalosporins.

Infections caused by ESBL-producing pathogens have been reported to be associated with risk factors such as age. In the current study, ESBL production was significantly higher in children (68.9%) than adults (31.1%) (p < 0.05). These findings compare well with those reported elsewhere [18,23].

## Conclusions

High prevalence of ESBL-producing *E. coli *and *Klebsiella spp *strains was found among inpatients and children. Most of the ESBL- producing isolates were multi-drug resistant making available therapeutic choices limited. We recommend continued antibiotic surveillance as well comprehensive multi-center studies to address the emerging problem of ESBL-associated infections in order to preserve the continued usefulness of most antimicrobial drugs. Further more conducting molecular studies will help to evaluate the various ESBL types.

## Competing interests

The authors declare that they have no competing interests.

## Authors' contributions

SM, SA, MK, EFL and SYM participated in the design of the study. SM oversaw the implementation of the study. SM, SA and MK were responsible for the laboratory testing. SM drafted the report and all co-authors participated in revising the report. All authors read and approved the final manuscript.
